# Targeting programmed cell death in metabolic dysfunction-associated fatty liver disease (MAFLD): a promising new therapy

**DOI:** 10.1186/s11658-021-00254-z

**Published:** 2021-05-07

**Authors:** Jianan Zhao, Yiyang Hu, Jinghua Peng

**Affiliations:** 1 Institute of Liver Diseases, Shuguang Hospital Affiliated to Shanghai University of Traditional Chinese Medicine, 528, Zhangheng Road, Shanghai, China; 2 Institute of Clinical Pharmacology, Shuguang Hospital affiliated to Shanghai University of Traditional Chinese Medicine, 528, Zhangheng Road, Shanghai, China; 3 Key Laboratory of Liver and Kidney Diseases, Ministry of Education, Shanghai University of Traditional Chinese Medicine, 528 Zhangheng Road, Pudong District, Shanghai, 201203 China; 4 Shanghai Key Laboratory of Traditional Chinese Clinical Medicine, 528, Zhangheng Road, Shanghai, China

**Keywords:** Metabolic dysfunction-associated fatty liver disease, Apoptosis, Necroptosis, Autophagy, Pyroptosis, Ferroptosis

## Abstract

Most currently recommended therapies for metabolic dysfunction-associated fatty liver disease (MAFLD) involve diet control and exercise therapy. We searched PubMed and compiled the most recent research into possible forms of programmed cell death in MAFLD, including apoptosis, necroptosis, autophagy, pyroptosis and ferroptosis. Here, we summarize the state of knowledge on the signaling mechanisms for each type and, based on their characteristics, discuss how they might be relevant in MAFLD-related pathological mechanisms. Although significant challenges exist in the translation of fundamental science into clinical therapy, this review should provide a theoretical basis for innovative MAFLD clinical treatment plans that target programmed cell death.

## Introduction

Metabolic dysfunction-associated fatty liver disease (MAFLD; also referred to as metabolic-associated fatty liver disease) has replaced nonalcoholic fatty liver disease (NAFLD) to more accurately reflect the mechanism seen in patients with metabolic dysfunction and fatty livers [1–5]. Therefore, MAFLD is used throughout this article.

MAFLD is a chronic disease that has become one of the most common reasons for liver transplantation, especially in Western countries [6]. The condition is closely related to metabolic disorders, including type 2 diabetes, hypertension, hyperlipidemia, and obesity. The current internationally accepted clinical practice guidelines state that a diagnosis of MAFLD should be based on histological (biopsy), imaging and/or blood biomarker evidence of fat accumulation in the liver (hepatic steatosis) along with one of the following criteria: obesity, confirmed type 2 diabetes mellitus (T2DM), or evidence of metabolic dysregulation [2, 7]. Other pathogenic factors and pathological mechanisms known to be involved in the complex disease progression of MAFLD include exercise, diet, intestinal microflora disorders, genetic susceptibility, fat accumulation, lipotoxicity, oxidative stress, endoplasmic reticulum stress, mitochondrial dysfunction, and intestine–liver axis-related signal transduction, among others [6].

Cell death occurs in various physiological and pathological processes in the body [8]. In specific diseases, it triggers distinct responses that are context-dependent [9]. The death of liver cells can trigger and aggravate chronic inflammation and liver fibrosis, advancing to chronic cirrhosis and liver cancer. While cell death in the liver releases a variety of pro-inflammatory cytokines and triggers the organ's regeneration responses, it also comes with the risk of acute liver failure. Chronic liver disease is indicative of an insufficient response to cell death, leading to ineffective wound healing, which increases the risk of fibrosis and hepatocellular carcinoma (HCC) [10, 11].

In vitro and in vivo experiments have demonstrated the key role of liver cell death in MAFLD. Liver cell damage, liver cell death, inflammation and oxidative stress, the principal pathogenic features of MAFLD, are interrelated [12]. In this review, we discuss possible forms of regulatory cell death (RCD) or programmed cell death (PCD) and their molecular mechanisms in MAFLD. The aim is to provide a theoretical basis for targeting RCD or PCD pathways as a novel treatment for MAFLD.

## Apoptosis

The concept of apoptosis was first proposed by Kerr et al.. Although it initially attracted little attention, excellent research progress has been made in the last ten years [13]. Apoptosis is considered a basic biological and physiological process. Its dysregulation is involved in various diseases and pathologies, including damage from drug toxicity, immune responses, infections and tumors, and metabolic disorders [14]. It has highly specific and recognizable morphological features. It is characterized by concentrated chromatin and cell shrinkage caused by DNA breakage. Under normal circumstances, the entire process of apoptosis is controllable, meaning that the integrity of the cell plasma membrane is maintained to prevent the leakage of cell contents, thus preventing inflammation [11].

## Mechanisms of apoptosis

**Fig. 1:**
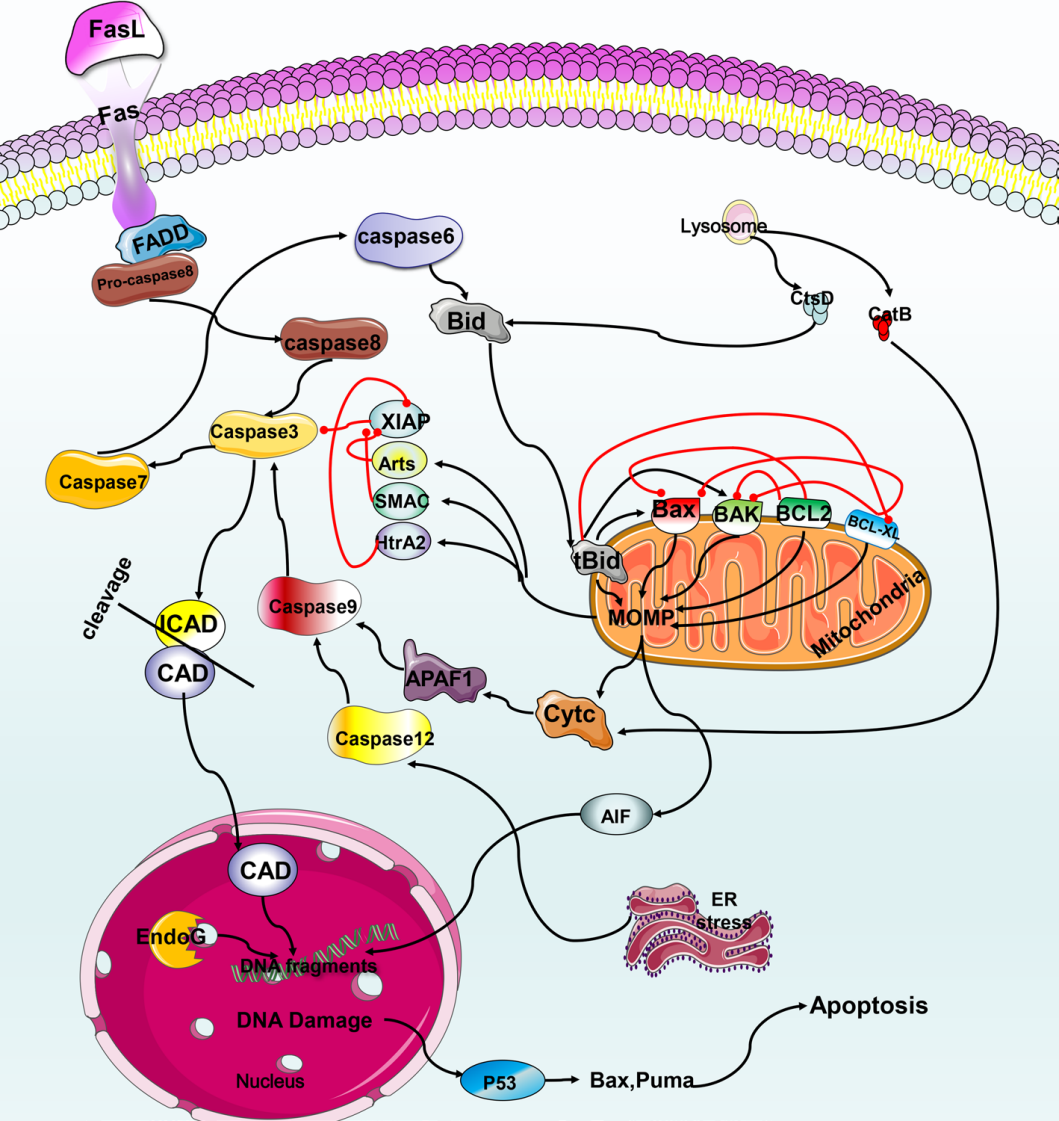
Mechanism of apoptosis. (1) After Fas and FasL are combined in the death receptor pathway, they recruit molecules, such as FADD, and activate caspase cascade reactions, including activation of caspase 8, caspase 3 and caspase 7. Caspase 8 activation can be blocked by C-FLIP. Then, caspase 3 and caspase 7 continue to cleave cell substrates, such as ICAD, and trigger apoptosis. (2) Caspase 3 and caspase 7 in the death receptor pathway may activate caspase 6 to cleave Bid into tBid, which is transferred to the mitochondria and induces the release of cytochrome c (cytc), AIF, Smac/DIABLO, Omi/HtrA2, etc. Cytc activates caspase 9, APAF1, etc. to cause apoptosis, and the other three inhibit the XIAP survival protein to promote apoptosis. (3) AIF translocation from the mitochondria to the nucleus may interact with EndoG to trigger DNA degradation and apoptosis. P53 induces apoptosis by transactivating the expression of various proapoptotic proteins. (4) Lysosomal permeabilization induces apoptosis through the release of ctsB and ctsD to activate the mitochondrial pathway. (5) Dysfunction of the endoplasmic reticulum leads to UPR-induced apoptosis by activating caspase 12, 9 and 3

Apoptosis primarily occurs the external death receptor pathway or the internal mitochondrial pathway. In addition, it can be triggered by mitochondrial dysfunction, lysosomal permeability, endoplasmic reticulum stress (ERS), and nuclear DNA damage [15] (see Fig. 1 for details).

**Fig. 2:**
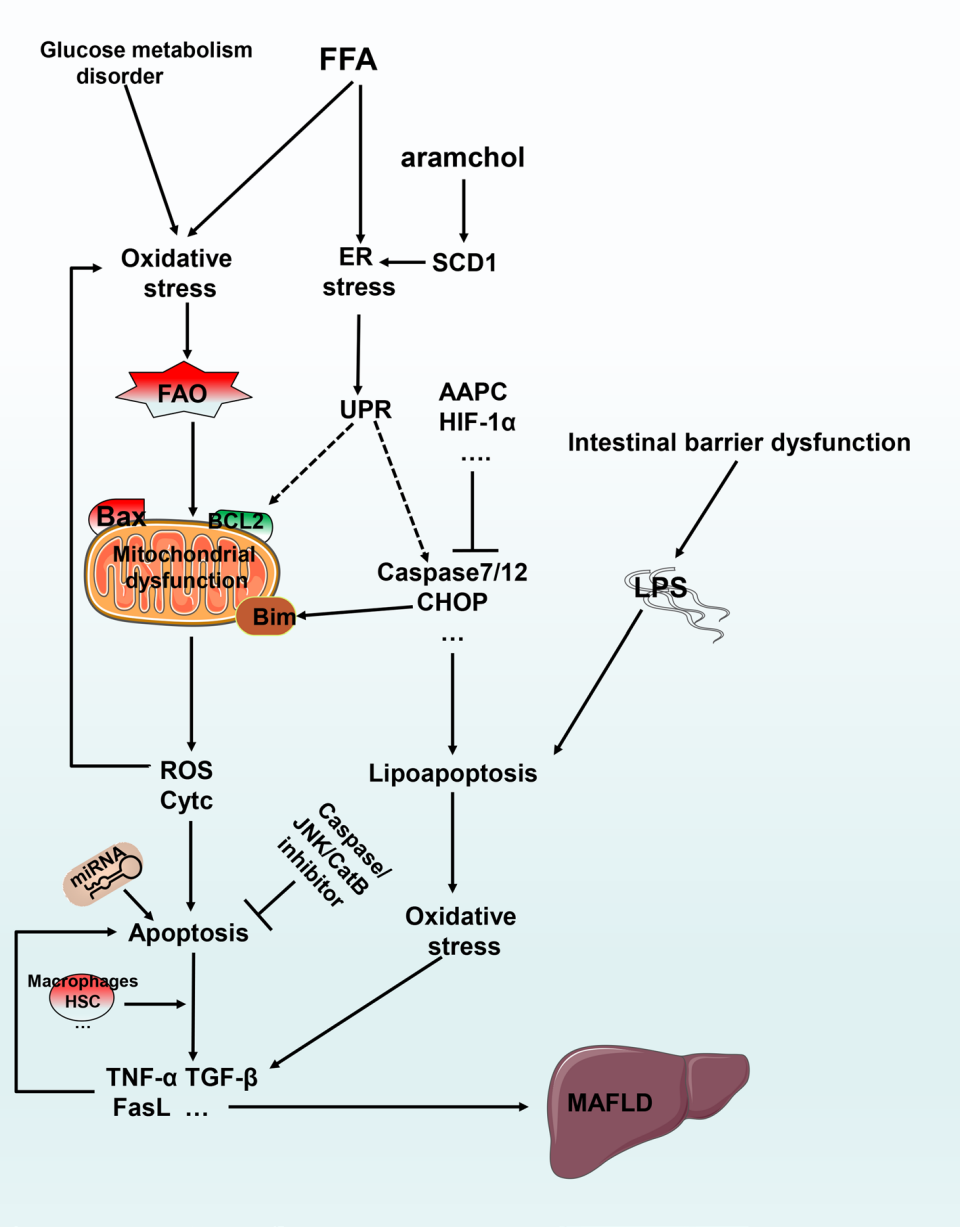
The relationship between apoptosis and MAFLD. Excessive FFAs and other compounds causes oxidative stress, ER stress and other causes of mitochondrial dysfunction, resulting in the release of ROS and cytochrome c with subsequent apoptosis. ER stress may cause lipoapoptosis through caspase 7/12 and other mechanisms, eventually leading to the development of MAFLD. MiRNAs can regulate apoptosis through different mechanisms. Intestinal barrier dysfunction causes LPS to activate lipoapoptosis

The external death receptor pathway is primarily mediated by members of the tumor necrosis factor (TNF) superfamily, such as Fas and the TNF receptor (TNFR). After activation by an exogenous ligand, a series of reactions lead to apoptosis (Fig. 2). Caspase 9 is the promoter of the intrinsic mitochondrial pathway and the B-cell lymphoma/leukemia-2 (BCL2) protein family mediates it. The BCL2 protein family can be divided into 3 subclasses. The first includes pro-apoptotic proteins, such as Bcl-2–associated X protein (Bax), BCL-2 homologous antagonist/killer (Bak), BCL-2 ovarian killer (Bok), BCL2-associated agonist of cell death (Bad), Noxa, and BCL-2 binding component 3 (PUMA). The second is anti-apoptotic proteins, such as BCl2 and BCL-XL (also known as BCL2L1). The third is the BH3-only protein, BH3-interacting domain death agonist (Bid), which is primarily responsible for crosstalk between the two apoptosis pathways. Bid is activated by caspase 6 to mediate mitochondrial outer membrane permeabilization (MOMP) and apoptosis [16].

## MAFLD and apoptosis

Liver biopsy remains the standard for MAFLD testing. Studies have shown that the levels of markers of hepatocyte apoptosis in plasma from patients with MAFLD correlate with histological severity. Therefore, the detection of activated caspase or the cytokeratin-18 (CK-18) fragment cleaved by caspase 3 are potentially noninvasive ways to detect human MAFLD and liver fibrosis [17]. However, when CK-18 levels, histology and liver fat contents were studied for a large multi-ethnic group of people, it was found that the degree of MAFLD could not be determined based on CK-18 [18]. Despite this, it remains useful when combined with other noninvasive methods, particularly because its levels are not affected by confounding factors, including age and obesity. Thus, it has the potential for clinical applications: further research is needed on population factors and the severity of fibrosis, among other things, to establish parameters for its use as a biomarker of MAFLD [18].

The accumulation of excess saturated fatty acids leads to apoptosis through oxidative stress and endoplasmic reticulum stress [19]. Hypertriglyceridemia, which results in lipoapoptosis, is found in non-obese MAFLD patients [20]. In fact, lipoapoptosis induced by free fatty acids is the primary form of apoptosis in MAFLD.

Lipoapoptosis enhances oxidative stress, which in turn increases the apoptosis, inflammation and fibrosis, creating a vicious cycle [21]. Egnatchik et al. found that primary hepatocytes and H4IIEC3 cells with palmitate and calcium chelators induced mitochondrial dysfunction by altering calcium homeostasis in the endoplasmic reticulum, which enhanced reactive oxygen species (ROS) production and apoptosis [22].

Lipoapoptosis and ERS are related, with the death receptor DR5 activated by ERS-related protein expression (CHOP) protein or by further binding c-Jun N-terminal kinase (JNK) to activate Bim (also known as BCL2L11), PUMA and other proteins to further activate caspase 3 [23]. Wang et al. experimentally demonstrated that Asiatic acid (AAPC) inhibits the expression of CHOP, caspase 12, JNK and other proteins, thereby attenuating ERS, apoptosis and lipid metabolism disorders [24].

In a recent clinical trial aramchol, a stearoyl-CoA desaturase 1 (SCD1) modulator, was used to treat MAFLD subjects for 3 months. Patient liver fat content was reduced and the treatment was well tolerated. Aramchol may regulate SCD1 fatty acid enzymes, ultimately affecting ERS and apoptosis [25, 26]. Recent studies showed that in a MAFLD mouse model, caspase 2 deletion reduced apoptosis and inhibited the profibrotic pathway, in turn inhibiting MAFLD progression [27]. Ferreira et al. evaluated subjects using the Kleiner-Brunt scale and found that the activation of caspases 3 and 2 and the DNA fragmentation in the liver of patients with severe MAFLD were significantly higher than in steatosis patients [28].

Intestinal barrier dysfunction in MAFLD leads to lipopolysaccharide (LPS) leakage of and disturbance of the intestinal flora, which may lead to excessive fat accumulation and lipoapoptosis [29]. Li et al. used LPS and free fatty acids (FFAs) to induce MALFD in vitro and found that the treatment upregulated Bax, cleaved caspase 3 and 8, and downregulated Bcl-2, leading to apoptosis, which was TNF-α- and caspase-dependent [30] (see Fig. 2 for details).

## MAFLD-related external death receptor pathway

Increased Fas expression is observed in patients with MAFLD [31]. Fas and FasL expressions and the rate of apoptosis are also higher in obese children with obstructive sleep apnea (OSA) than in obese children without OSA [32]. A study of MAFLD patients diagnosed by liver biopsy found that their CK-18 and TNF-α levels were higher than those of the control group [33]. The expressions of external death receptor pathway-related proteins are higher in MAFLD patients, and the use of relevant drugs improves disease-related indicators by inhibiting these proteins. Studies have found that transaminase levels, insulin resistance, adiponectin levels, liver fibrosis and steatosis improve in MAFLD patients when TNF-α levels are attenuated [34]. Furthermore, inhibition of Fas reduces hepatocyte apoptosis and reduces associated liver damage [35]. In addition, Kroy et al. found that deletion of c-Met in the liver cells of mice fed a methionine- and choline-deficient (MCD) diet led to upregulation of fatty acid metabolism genes. The increase in TUNEL-positive cells and superoxide anions aggravates the progression of MAFLD, while knockout of caspase 8 inhibits progression [36].

Interestingly, Physi et al. found that gastric bypass surgery (Roux-en-Y gastric bypass, RYGB) yields similar improvements in disease-related indicators as can be achieved with apoptosis-inhibiting drug therapy,and the levels of glucose-regulated protein-78 (Grp78), X-box binding protein-1 (XBP-1), spliced XBP-1, fibroblast growth factor 21 (FGF21), other ERS-related proteins and excessive apoptosis were all reduced compared to the control group [37]. Aerobic exercise improves MAFLD by inhibiting TNF-α and reducing ROS and cytochrome c levels [38].

## MAFLD-related internal mitochondrial apoptosis

The internal mitochondrial apoptosis pathway is also activated by the abovementioned factors. Resistance to lipoapoptosis in MAFLD is partly due to the existence of a hedgehog autocrine survival signaling pathway [39]. Geng et al. used Smad4 knockout mice to demonstrate the protective effect of Smad4 deletion on MAFLD: it blocks the mitochondrial apoptotic pathway by inhibiting expression of the pro-apoptotic genes Bax and caspase 3 [40].

Disorders in glucose metabolism in MAFLD can easily lead to hyperinsulinemia and hyperglycemia. Hyperglycemia can cause increased oxidative stress and trigger mitochondrial dysfunction, including mitochondrial depolarization, cytochrome c release, and changes in Bax and Bcl-2 expression. Recent studies have shown that casein kinase 2-interacting protein-1 (CKIP-1) may regulate insulin signaling by inhibiting the phosphorylation of JNK1. The CKIP-1-deficient MAFLD model exhibits more severe fatty liver through an increase in phosphorylated JNK1, which further inhibits insulin receptor substrate-1 (IRS-1) serine phosphorylation and IRS-1 tyrosine phosphorylation, ultimately aggravating insulin resistance, hyperglycemia, apoptosis and MAFLD [41]. Cyanidin-3-O-β-glucoside (C3G) inhibits the caspase 3, caspase 9, Bax and JNK pathways by reducing hyperglycemia-induced oxidative stress and related mitochondrial disorders to improve MAFLD [42]. Other, similar studies indicate that intermittent hyperglycemia (IHG) in the setting of lipotoxicity may lead to oxidative stress and hepatocyte apoptosis by increasing mitochondrial permeability transition (MPT) and mitochondrial dysfunction, which promotes MAFLD [43].

## MAFLD-related miRNAs regulate apoptosis

Analysis of various MAFLDs have enabled researchers to determine multiple miRNAs with expression changes related to the condition. It is believed that there may be therapeutic potential in regulating apoptosis through relevant miRNAs [44, 45]. Castro et al. identified a relationship between the miR-34a–SIRT1–p53 pro-apoptotic pathway and hepatocyte apoptosis: miR-34a, apoptosis and acetylated p53 levels increased in MAFLD livers, while the SIRT1 level decreased. This pathway is specifically regulated by ursodeoxycholic acid (UCDA) [46]. One study found that after treatment with creatine, the miR-34a–SIRT1–p66shc anti-apoptotic pathway may reduce high-fat diet or palmitic acid-induced increases in cleaved caspase 3, caspase 3 and caspase 9, thereby improving MAFLD [47]. MiR-296-5p and miR-615-3p regulate lipid-associated apoptosis levels in MAFLD by negatively regulating PUMA, a pro-apoptotic protein, and CHOP [48, 49]. Notably, miR-21 may play a distinct role in MAFLD. Rodrigues et al. found that combined ablation of miR-21 with obeticholic acid improves MAFLD-related pathology, including steatosis, inflammation and lipoapoptosis [50].

## Necroptosis

Necroptosis was first observed in experiments by Ray et al. in 1996 [51]. It is characterized by morphological changes similar to those observed in necrosis, but is distinct in that it is a controllable form of death. Its morphological changes include organelle swelling, damage to the plasma membrane and release of cellular contents, which may lead to the occurrence of secondary inflammation [12]. Key molecules for necroptosis include mixed lineage kinase domain-like (MLKL), and RIP protein kinase family members RIPK1 and RIPK3.

**Fig. 3:**
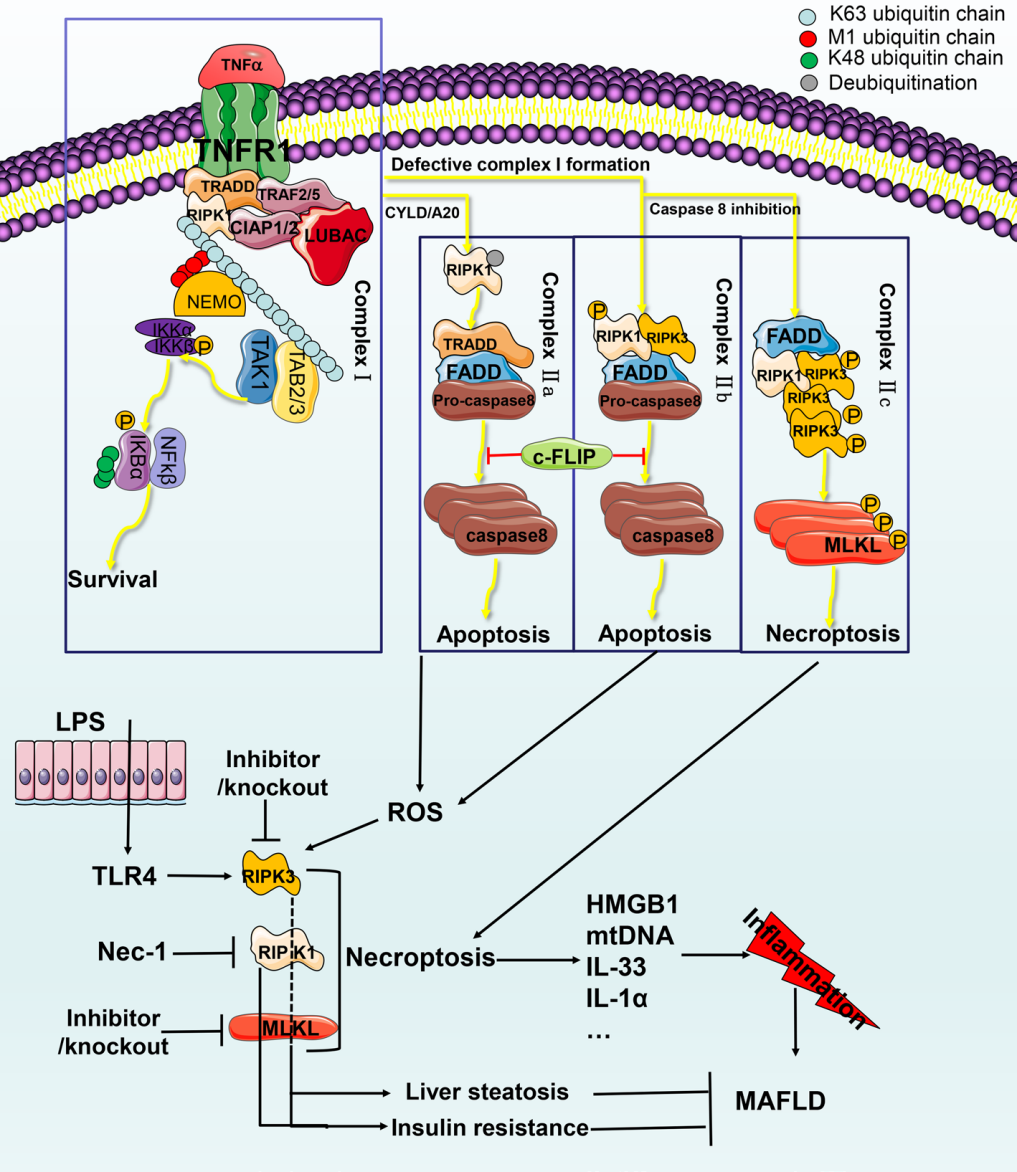
Mechanism of necroptosis and its relationship with MAFLD. (1) TNF activates TNFR1 and recruits TRADD, TRAF2/5, clAP1/2 and LUBAC to form complex I. RIPK1, TAB2/3, Clap1/2 and LUBAC undergo M1 and K48 ubiquitination events and connect and recruit NEMO. IKKa, IKKb and RIPK ubiquitination events lead to the formation of the TAK complex, which phosphorylates IKK2 and activates the NF-KB pathway, cFLIP protein formation, K48 phosphorylation events and proteasome degradation. (2) CYLD or A20 deubiquitinates RIPK1 and induces dissociation of TRADD and RIPK1 to form complex IIa, including FADD, pro-caspase 8, and TRADD. Complex IIb includes RIPK1/3, FADD, and caspase 8. cFLIP inhibits complex IIa and IIb-induced apoptosis. (3) When caspase 8 is inhibited, complex IIc (the necrosome) is formed, containing RIPK1, RIPK3 and MLKL. RIPK1 phosphorylates RIPK3 and activates MLKL to cause changes in cell membrane permeability and necroptosis. The released substances cause inflammation. Activated TLR4 and ROS-activated RIPK3 may be related to liver fibrosis and insulin resistance. Inhibition of RIPK1 and MLKL may improve liver steatosis and insulin resistance

TNF is a key cytokine in inflammation and other aspects of biology. TNFR1 has been widely studied in regulating cell survival, apoptosis and necroptosis [52]. TNFR1-mediated signal transduction is an example of the molecular mechanism of necroptosis and the conversion between apoptosis and necroptosis (see Fig. 3 for details).

## MAFLD and necroptosis

The destruction of membrane integrity during necroptosis releases various proinflammatory mediators and promotes the progression of MAFLD [53]. Ding et al. found that AS1842856 improves MAFLD by inhibiting forkhead box protein O1 (FOXO1), ERS and necroptosis [54]. TNF-α-mediated necroptosis is widely recognized as the most classical pathway. Both human MAFLD samples and experimental animal MAFLD livers can show elevated TNF-α [55] and TNFR1 [56] levels. In addition to TNF-α, other pattern recognition receptors, such as toll-like receptor 4 (TLR4), are also involved in the activation of necroptosis. In MAFLD, hepatocytes are exposed to a large number of TLR4 ligands, such as gut-derived LPS [57], which subsequently activate the TLR4 receptor. It activates RIPK3 and MLKL, which trigger necroptosis [58].

## RIPK3 in MAFLD

RIPK3 kinase is an indispensable component of necroptosis. Studies have shown that the expression levels of RIPK3 correlate with the sensitivity of cells to necroptosis and are very low in the liver under normal physiological conditions. This indicates that under normal circumstances, necroptosis does not frequently occur in the liver and may be a back-up programmed death regulatory mechanism when apoptosis fails [59–61].

Human MAFLD liver samples show strong upregulation of RIPK3 [62]. Gautheron found that RIPK3 mediates MAFLD development through a positive feedback loop with JNK that includes liver cell death, inflammation, and fibrosis [63]. Based on results for various experimental MAFLD animal models, the role of RIPK3 appears to be context-dependent. The lack of RIPK3 in high-fat diet experiments by Roychowdhury et al. showed that increased liver damage, liver steatosis, ALT/AST, inflammation and apoptosis may occur due to inhibition of necroptosis, which converts the form of programmed cell death to apoptosis, exacerbating damage [64]. Saeed et al. also used a high-fat diet to induce a model of RIPK3 knockout, which aggravated liver steatosis but partially inhibited inflammation [65]. Due to the complexity of RIPK3, it seems that direct targeted inhibition of RIPK3 kinase is not an ideal strategy for MAFLD therapy. Further research is needed to clarify the role of RIPK3, and other key molecules should be examined as well, including RIPK1 and MLKL.

## RIPK1 and MLKL in MAFLD

Studies have shown that RIPK3 self-oligomerization is sufficient to induce necroptosis. RIPK1 kinase acts as a positive regulator of RIPK3 by forming a amyloid-like oligomers with RIPK3. It can also act as a negative regulator of RIPK3 in cells to promote cell survival [66]. Studies have shown that using RIAP-56, a potent inhibitor of RIPK1, improves the histological characteristics of MAFLD in HFD mice through MLKL, reducing liver inflammation, fibrosis and liver lipid accumulation. Its mechanism may involve ameliorating mitochondrial dysfunction and promoting β oxidation [67].

In addition, MLKL deficiency may be regulated by phosphatidylinositol (3,4,5)-trisphosphate (PIP3) in liver cells, which reduces insulin resistance and glucose intolerance. Paradoxically, it was found that neither MLKL nor RIPK1 inhibition reduced inflammation [68]. Saeed et al. found that patients with MAFLD exhibited increased MLKL levels compared to the non-MAFLD group and that MLKL^−/−^ mice induced with a high-fat diet showed decreased transferase levels, triglycerides, MAFLD activity scores, steatosis score, inflammation, balloon degeneration, and expression of de novo lipogenesis (DNL) genes in the liver [69]. However, Suda found that RIPK1 antisense knockdown caused α-galactosylceramide-treated C57BL/6 mice to undergo large-scale apoptosis-type immune liver injury. Lethality and was not associated with the inhibition of NF-kB and necroptosis [70].

Most current research involves knockout or inhibition of key molecules of systemic necroptosis in disease models. Therefore, further studies on pure liver-specific knockout and immune system-related connections are needed.

## Autophagy

The research history of autophagy spans several decades. It was first officially named autophagy by de Duve, who was building on earlier discoveries [71–73]. The name derives from the Greek roots *phagía* = eat or consume and *auto* = self [74]. Cell autophagy involves the formation of autophagosomes (bilayer membrane vesicles that originate as omegasomes on the endoplasmic reticulum) and autophagolysosomes. Their function is to recover and reuse unwanted cellular proteins [75, 76], carbohydrates [77] and lipids [78]. Autophagy is an important form of death that controls the degradation of intracellular waste and efficiently recycles substances.

## Molecular mechanisms of autophagy

**Fig. 4:**
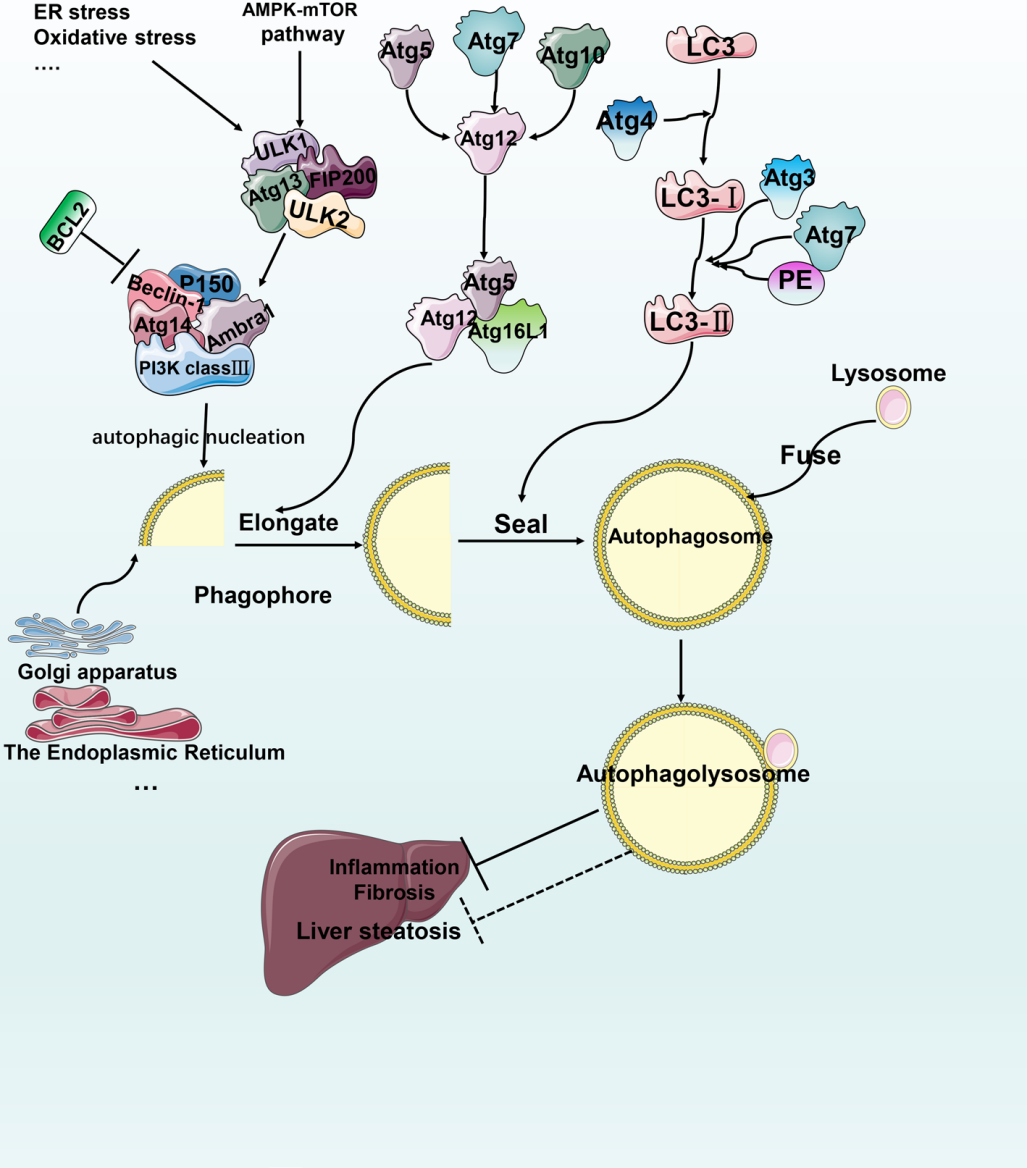
Mechanism of pyroptosis. (1) In the classical pathway, PAMPs/DAMPs activate assembly of the NLRP3 complex and sequentially cleave and activate caspase 1 to cause the maturation and release of IL-1β and IL-18 or to activate gasdermin to form a pore structure that leads to pyroptosis. (2) In the nonclassical pathway, activation of caspase 4, 5 and 11 cleaves GSDMD and causes pyroptosis. The two pathways engage in crosstalk with each other via panxinin-1 and GSDMD

Selective autophagy can be targeted to degrade functionally damaged or excessive organelles, microorganisms, lipids, etc. [79]. There are two key steps in the metabolic autophagy pathway in the liver: formation and degradation (autophagic flux). Three major forms of autophagy coexist: macroautophagy, chaperone-mediated autophagy and microautophagy. For more detailed molecular mechanisms, see the extensive reviews on autophagy [80, 81]. Herein, we primarily focus on macroautophagy (hereinafter referred to as autophagy) and its selective forms (see Fig. 4 for details).

## MAFLD and autophagy

Under physiological conditions, autophagy is primarily induced by starvation. It maintains the body's metabolic homeostasis by regulating biochemical reactions, such as gluconeogenesis and fatty acid oxidation [82]. The liver is one of the principal vital organs of metabolism. Studies show that starvation stimuli activate autophagy in the liver peaks in just a few hours [83].

Autophagy is indispensable for liver metabolism and has been considered to have a protective effect on the liver. When mice lack key Atg proteins of autophagy, liver cells are more susceptible to damage [84]. MAFLD patients often have a high-fat diet. Short-term high-fat intake will activate autophagy to prevent the occurrence of lipotoxicity. However, long-term chronic high-fat intake restricts autophagosome and lysosome fusion, increasing the risk for MAFLD development [85].

## Autophagy and lipid metabolism in MAFLD

The selective form of autophagy for fat is referred to as lipophagy [86]. Under physiological conditions, it primarily works through the synergistic effect of cytosolic and lysosomal lipases and the lipid droplet transport of RAB7 to engulf lipids and increase free fatty acid content. In pathological conditions, impaired autophagy can lead to fat accumulation. Ubiete-Franco et al. found that the levels of glycine N-methyltransferase (GNMT) in patients with MAFLD were reduced and that GNMT knockout mice exhibited increased levels of methionine and its metabolite S-adenosylmethionine (SAMe) along with inhibited autophagic flux through methylated PP2A, which may be one of the mechanisms leading to increased liver fat production [87]. Byun et al. found that phosphorylation of Jumonji-D3 (JMJD3) at Thr1044 by FGF21 signal-activated PKA increases its nuclear localization and interaction with the nuclear receptor PPARα to transcriptionally activate autophagy genes, such as Tfeb, Atg7 and Atgl, causing lipolysis. It also reduces the liver expression of JMJD3, Atg7, LC3 and ULK1 in MAFLD [88]. Wang et al. found that formononetin causes lipophagy to reduce fat accumulation by activating AMPK and promoting nuclear translocation of TFEB in HFD mice [89]. Tang et al. found that osteopontin (OPN) was elevated in an MAFLD mouse model and that this, combined with integral αVβ3 and αVβ5, can reduce FFA-induced autophagy in HepG2 cells, leading to lipid accumulation, which is reversed by inhibiting OPN [90]. Some traditional Chinese herbal medicine seems to have advantages in treating MAFLD with autophagy as the target [91, 92]. Ren et al. used catalpol, an iridoid glucoside derived from the rehmannia root, to improve hepatic steatosis in ob/ob fatty liver mouse models induced by a high-fat diet, and the authors speculated that catalpol’s anti-fat denaturation may enhance nuclear translocation of TFEB through phosphorylation activation of AMPK [93].

However, there are also studies showing that impaired autophagy reduces fat production. For example, when autophagy in the mouse liver is impaired, triglyceride levels decrease and ketone body production is impaired [94].

AMPK/mTOR-regulated autophagy may represent a target for MAFLD. Tong et al. found that PPARα regulates autophagy through the AMPK/mTOR pathway, reduces intrahepatic fat and stimulates β-oxidation in liver cells [95]. Zeng et al. found that acetylshikonin (AS) improves MAFLD and may increase liver autophagy levels through the AMPK/mTOR pathway [96].

## Autophagy, metabolic stress and insulin resistance in MAFLD

Obesity models often exhibit decreased expression of the ATG7 protein. Impaired autophagy is usually accompanied by ER stress and insulin resistance. The latter leads to elevated insulin, which will in turn aggravate autophagy dysfunction [97, 98]. This may be controversial because Yan et al. found that chlorogenic acid inhibits autophagy through the JNK pathway to reduce insulin resistance and improve MAFLD [99].

Zhang et al. found that increased levels of P62 and LC3-II in experimental MAFLD mice indicate the inhibition of autophagy and cause increases in GRP78, PDI, p-PERK, p-eIF2a and eIF2a, indicating the emergence of ER stress [100]. Similarly, Lee et al. found that *Eucommia ulmoides* leaf extract improves both autophagic flux and HFD-induced steatosis in mice by inhibiting mTOR and the ER stress-related proteins PERK, p-eIF2a, GRP78 and CHOP [101]. Unconventional activation of sterol regulatory element-binding protein 2 (SREBP-2) leads to MAFLD cholesterol accumulation. Deng et al. found that inhibition of SREBP-2 reduces ERS by enhancing the LC3-II-to-LC3-I ratio, autophagic flux and lipolysis, and inhibiting PERK-P-EIF2α signaling [102]. However, Kim et al. reported that administration of lovastatin and ezetimibe increased SREBP-2 in HFD mice and resulted in the interaction of patatin-like phospholipase domain-containing enzyme 8 (PNPLA8) with LC3 to promote autophagy and reduce hepatic steatosis [103].

Additionally, Jiang Zhi granules (JZGs) inhibit palmitate-induced autophagosome flux damage and promote the fusion of autophagosomes and lysosomes to restore autophagy, protecting liver cells from oxidative stress damage and mitochondrial disorders [104]. Irbesartan inhibits PKC and activates AMPK and ULK1, increasing the number of autolysosomes and autophagosomes. Upregulation of the autophagy proteins Atg5 and LC3BII/I reduces lipid deposition, improving mitochondrial function and reducing ROS levels [105].

## Autophagy and MAFLD-related liver fibrosis

Lipid bilayer stress can induce ER stress and control autophagy to mediate the steady state of the unfolded protein response (UPR) via the IRE-1–XBP-1 axis in MAFLD [106]. XBP1-mediated UPR activates HSCs to secrete collagen 1-α in a TGF-β-independent manner through autophagy. This effect is disrupted by inhibiting autophagy [107]. Transforming growth factor-β activated kinase-1 (TAK1) can be triggered in response to different cytokines, including IL-1, TNF-α and TGF-β. When TAK1 is enabled, it enhances autophagic activity by inhibiting mTORC1 activity and the AMPK pathway. The authors found that mTORC1 activity was enhanced, while AMPK and autophagy were inhibited, in TAK1-deficient mice, leading to spontaneous liver fibrosis and even cirrhosis. The inhibition of mTORC1 reversed the abovementioned phenotype [108].

It is useful to note that the role of autophagy in MAFLD-related fibrosis may have two sides. Autophagy can engulf lipid droplets in HSCs through lipid phagocytosis, providing energy substrates, such as ATP for HSC trans differentiation, which ultimately ameliorates liver fibrosis. Furthermore, autophagy-deficient cells exhibit lower HSC activation rates [109, 110].

## Others

In addition to the three PCDs mentioned above, there may be other PCDs in MAFLD. Because the overall experimental evidence on their potential functions in MAFLD seems not so advanced, we recommend that researchers continue to conduct in-depth investigations of these pathways.

## Pyroptosis and MAFLD

Pyroptosis is a newly discovered form of programmed death that is characterized by cellular content release mediated by caspases. It was officially named pyroptosis from the Greek roots *pyro* and *ptosis* to reflect its proinflammatory activity [111].

**Fig. 5:**
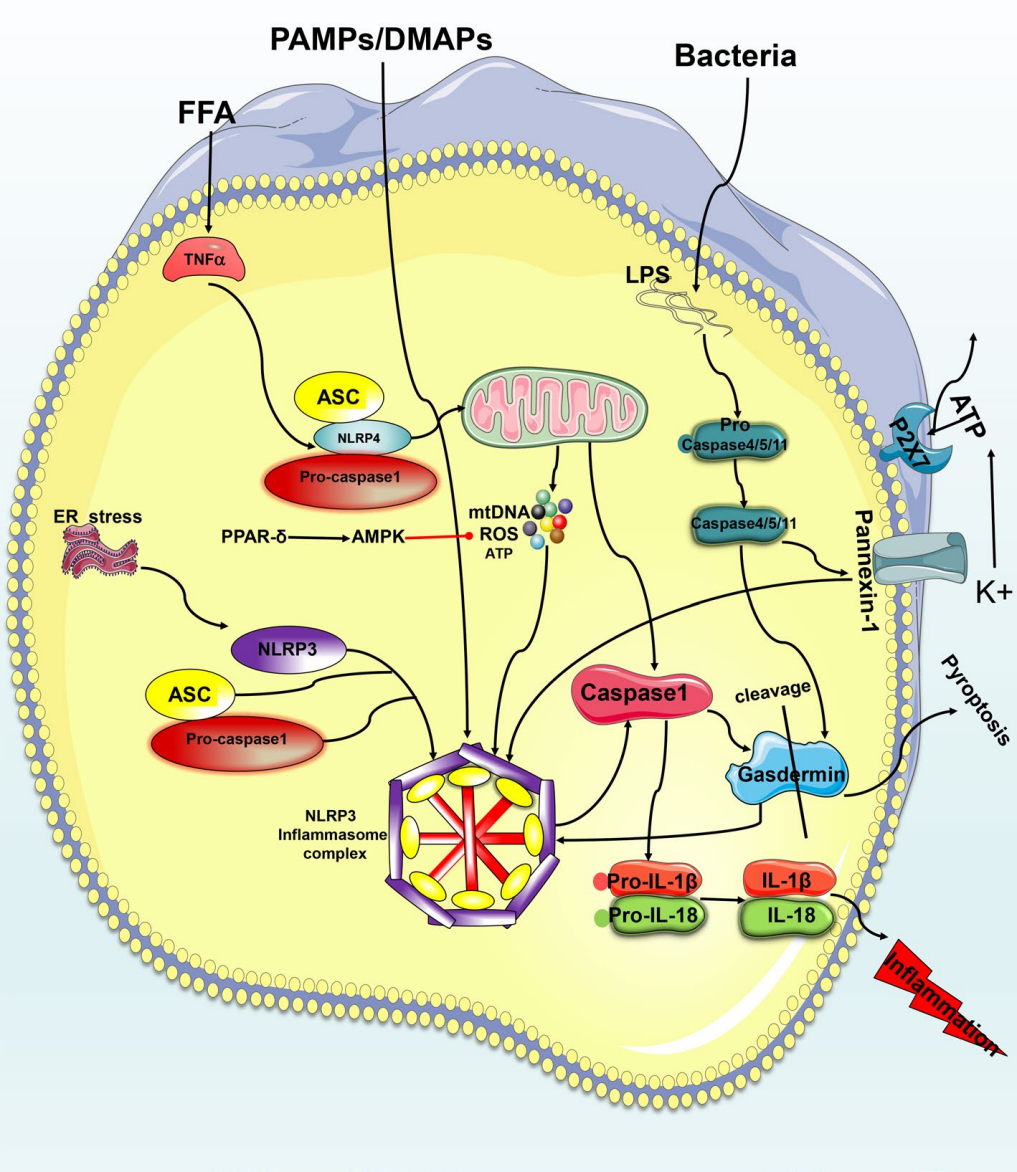
The molecular mechanism of ferroptosis and its relationship with MAFLD. The Fenton reaction and lipoxygenase regulation of ferroptosis via three regulatory pathways. Inhibition of the xc-system leads to depletion of GSH and excessive iron deposition through the Fenton reaction, causing lipid peroxidation and further leading to the progression of MAFLD

The morphological characteristics of pyroptosis are distinct from other forms of programmed death. It involves rapid rupture of the plasma membrane and the release of proinflammatory intracellular contents. Nuclear DNA lysis also occurs in pyroptosis, similarly to apoptosis [112]. Pyroptosis involves caspases 1, 4, 5 and 11 sensing different pathogen-associated molecular pattern (PAMP) or damage-associated molecular pattern (DAMP) activation and forming a pore-like structure on the cell membrane. Leakage of inflammatory mediators, such as IL-18/1β, leads to cell lysis and death [113]. Pyroptosis may occur through the classic caspase 1-dependent pathway [114] and the nonclassical caspase 4/5 (mouse caspase-11) pathway [115]. Its key molecules include NOD-LRR and pyrin domain-containing 3 (NLRP3); gasdermin D (GSDMD); and caspase 1, 4, 5 or 11 (see Fig. 5 for details).

Recent research suggests that pyroptosis represents a key link to MAFLD [116]. Zhong et al. found significant pyroptosis in a mouse model of MAFLD induced by HFD and a MAFLD model in liver cells induced by FFA. Genipin(GNP)reduced the expressions of pyroptosis-related genes and release of lactate dehydrogenase by inhibiting uncoupling protein-2 (UCP2).Overexpression of UCP2 upregulated the degree of pyroptosis. Finally, they proved that GNP, a natural water-soluble cross-linking agent, alleviates MAFLD by inhibiting UCP2-mediated pyroptosis [117].

Inhibiting activation of the NLR family to mediate pyroptosis may be a potential therapy for MAFLD. A large induction of NLRP3 activates MAFLD [116]. Studies have shown that berberine has a therapeutic effect on MAFLD through inhibition of the ROS–TXNIP axis, NLRP3, caspase 1 activity, and GSDMD-N expression [118]. In addition to NLRP3, the NLR family members NOD-LRR and pyrin domain-containing 4 (NLRP4) are also related to MAFLD. Recently, Chen et al. constructed a MAFLD cell model in vitro and found that NLRP4 is regulated by TNF-α levels and can be ectopically transferred to the mitochondria after activation by free fatty acids, and the rest of the process was similar to NLRP3.

Caspase 1 activation and lysing induces expression of IL-18 and IL-1β, eventually leading to pyroptosis and the release of proinflammatory cytokines [119]. It is worth noting that the IL-18 and IL-1β released by MAFLD during pyroptosis seem to be a double-edged sword [113]. Studies by Yamanishi et al. have demonstrated that IL-18 is essential for normal lipid metabolism in the liver and that its deficiency causes fat accumulation [120]. However, IL-1β is considered a pro-inflammatory cytokine that promotes the development of MAFLD. Studies have shown that GSDMD and GSDMD-N levels are higher in human MAFLD liver samples and are related to the MAFLD activity score (NAS) and fibrosis, while GSDMD knockout in the MCD diet-induced MAFLD model relieves MAFLD and fibrosis by reducing NF-KB activation and proinflammatory cytokines, such as TNF-α, IL-1 and MCP-1 [116].

Ezquerro et al. found that inhibition of TNF-α-induced apoptosis, autophagy and pyroptosis may exert protective effects against MAFLD [121]. Wang et al. discovered that GSDME converts caspase 3-mediated apoptosis into pyroptosis [122].

## Ferroptosis and MAFLD

Ferroptosis was first observed in experiments by Dolma et al. in 2003 [123]. Until 2012, the oncogenic RAS-selective lethal small molecule erastin, which was observed in experiments by Dixon et al., was known to induce this form of death, which is distinct from apoptosis [124].

**Fig. 6:**
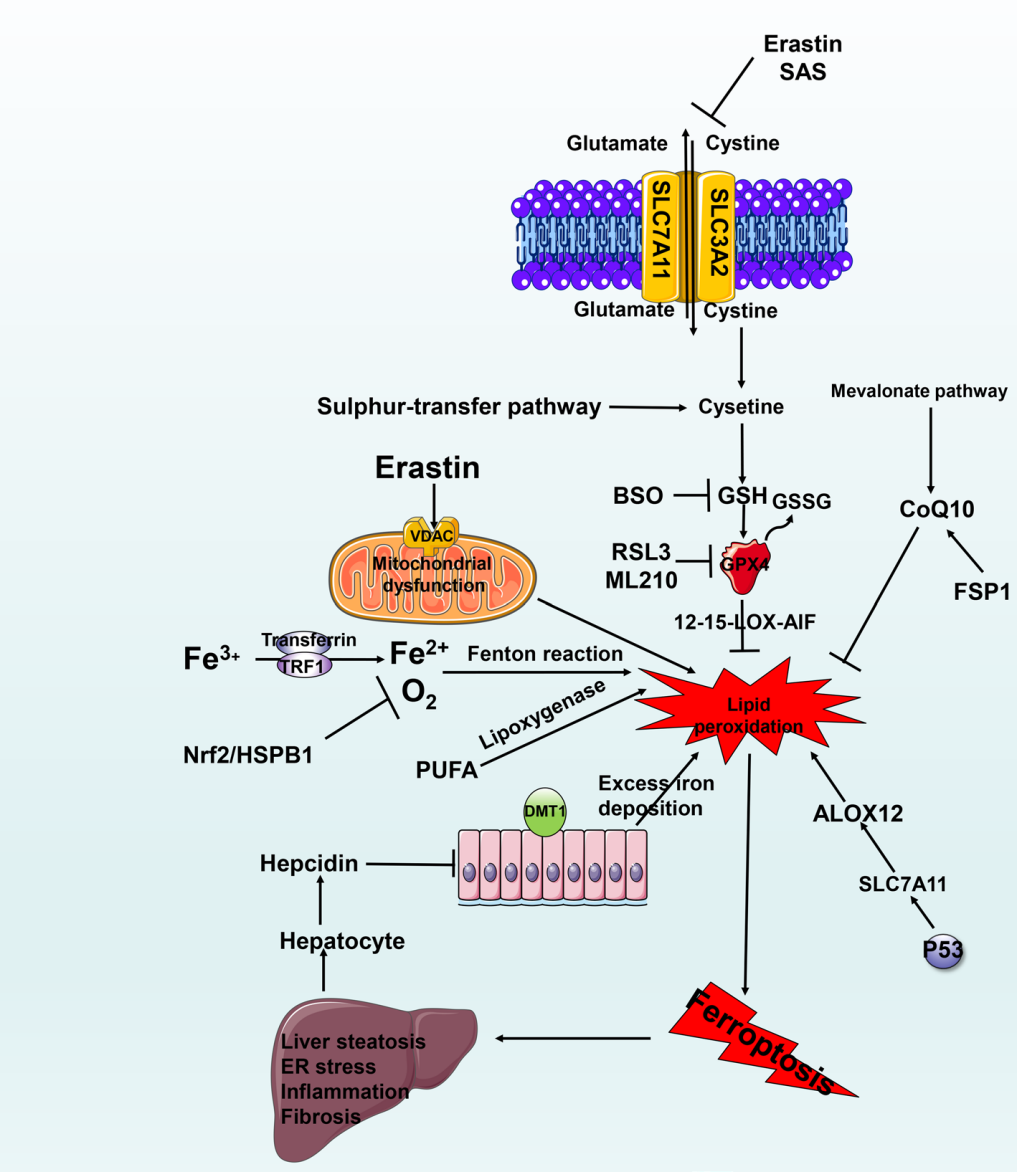
The molecular mechanism of autophagy and its relationship with MAFLD. (1) Under normal conditions, mTORC1 phosphorylates and combines with ULK1/2, mAtg13 protein and FIP200 protein to form a functional silencing complex. When ER stress, oxidative stress, etc. activate autophagy, the functional silencing complex dissociates, and autophagosomes begin to recruit Atg protein, ULK1/2, mAtg13 and FIP200, to form a functional complex. (2) The autophagy-inducing complexes PtdIns3K class III, hVps34, Beclin1, and P150 are transported to the ER through microtubules to begin autophagic nucleation to induce the formation of restricted membranes. (3) Two ubiquitin-like conjugation systems, Atg12-Atg5-Atg16 L and PE/LC3, form a complex and participate in membrane elongation and isolate membrane-formation events to finally close into a double-layer membrane vesicle structure. (4) The fusion of autophagosomes and lysosomes leads to cargo degradation. The degraded products (amino acids, etc.) are then released into the cytosol through the lysosomal membrane permease, which produces a subsequent response that may be related to MAFLD-induced lipid accumulation, fibrosis and inflammation

Morphologically, ferroptosis is characterized by cytological changes that include reduced cell volume and increased mitochondrial membrane density [125]. It is characterized by iron dependence and lipid peroxidation. It involves the pharmacological cross-regulation of l-glutathione (GSH) and glutathione peroxidase 4 (GPX4). The classical pathway primarily includes enzymatic reactions (the lipoxygenase pathway) and nonenzymatic reactions (the Fenton reaction) [126]. System xc-regulated GSH production through glutamate–cysteine transport inside and outside the cell. GPX4 is one of the GPXs with antioxidant activity. It can remove lipid peroxides formed by polyunsaturated fatty acid phospholipids with the help of system xc-regulated glutathione content, thereby reducing lipid peroxidative damage in the cell membrane [127, 128] (see Fig. 6 for details).

Therefore, classic ferroptosis inducers (FINs) are divided into two categories. Class I inducers are primarily systemic xc inhibitors, which are characterized by reduced synthesis of GSH and exhaustion of GSH content, leading to damage to the antioxidant system, peroxidative damage and ferroptosis [129]. Class I includes erastin and SAS. Class II inducers, such as RSL3 and ML210, directly inhibit GPx4 activity to trigger ferroptosis [130].

The two primary regulatory pathways of ferroptosis are the mevalonate pathway (primarily regulates GPX4 through isopentenyl pyrophosphate (IPP), which stabilizes the selenocysteine-specific tRNA) [131] and the sulfur-transfer pathway (regulates the body’s methionine, and sulfur-containing amino acid levels to ensure conversion to cysteine to synthesize GSH to help GPX4 regulate ferroptosis) [132], although there are others [133]. More detailed molecular mechanisms can be found in reviews on these topics [133].

Whether ferroptosis occurs depends on the balance between the accumulation of iron ROS and the body’s antioxidant system. When hydrogen atoms are extracted from unsaturated fatty acids, lipid peroxidation reactions begin, including a destructive chain reaction that produces heterogeneous groups of lipid peroxides [134], eventually resulting in cell dysfunction and the production of malondialdehyde (MDA) and 4-hydroxy-2,3-nonanal (4-HNE). 4-HNE was noted in the cytoplasm to disrupt liver cells in MAFLD patients through the Fenton reaction [135]. MDA and 4-HNE are elevated in 90% of MAFLD patients [136]. Imai et al. suggested that GPX4 antioxidant properties may play a key role in MAFLD [137]. Carlson et al. found that mice with specific deletion of the GPX4 gene in hepatocytes died during the embryonic stage and had extensive hepatocyte degeneration [138]. Combined with the results of studies by Kim et al., who found high regulation of Gpx4 in the liver [139], these findings suggest a correlation between ferroptosis and MAFLD. In addition, the use of RSL-3, a GPX4 inhibitor, may affect lipid peroxidation in the liver through a 12/15-Lox-AIF-related pathway. 12/15-LOX activation aggravates endoplasmic reticulum stress, inflammation, liver steatosis, and liver damage, and MAFLD is improved by the use of iron chelators [140].

Studies have found that liver iron deposition in MAFLD patients positively correlates with histological severity and can lead to the development of MAFLD [141]. Both lipid peroxidation and excess iron deposition may exacerbate MAFLD through ferroptosis. Fe^3+^ found in people’s daily diets is reduced to Fe^2+^ and then absorbed by the divalent metal transporter 1 (DMT-1) protein in the small intestine and stored in the intestinal cells or excreted outside the cell base. It is subsequently transported out through ferroportin and further reoxidized to Fe^3+^ by hephaestin [142]. In this process, hepatocytes control the production of hepcidin by sensing the iron content in the body. Hepcidin can reduce the expression of DMT-1 and thus reduce the intestinal absorption of iron [143]. In patients with MAFLD, hepcidin levels in the serum and in white fat were accompanied by upregulation of DMT-1 [144]. Upregulation of transferrin receptor 1 (TRE1) was also detected in a fatty liver mouse model [145]. TRE1 is a receptor that binds transferrin and is expressed on activated hepatic stellate cells (HSCs). It aids ferroptosis by increasing iron intake and reducing iron output through transferrin [146]. Excessive iron content deposition promotes liver fibrosis through lipid peroxidation. Ramm et al. identified a relationship between iron load and the activation of HSCs. Iron loading increased the numbers of activated HSCs and collagen deposition [147].

## Crosstalk between different PCDs in MAFLD

As mentioned earlier, apoptosis and necroptosis transform each other. Wang et al. discovered that GSDME converts TNFα-induced apoptosis into pyroptosis [122]. Bcl2 anti-apoptotic proteins can inhibit autophagy, whereas other pro-apoptotic proteins can promote autophagy [148]. Mitophagy is a form of autophagy that selectively clears damaged mitochondria. When damaged or inhibited, the failure to clear damaged mitochondria results in mitochondrial dysfunction, which produces excessive ROS and causes NLRP3 activation of pyroptosis. It promotes the production of pro-inflammatory factors to create a pro-inflammatory environment [149]. The activation of autophagy can be considered anti-inflammatory, possibly through inhibition of the activation of NLRP3 [150] and control of mitochondrial homeostasis [151]. Qiu et al. found that arsenic trioxide (AsO) induces MAFLD in mice, accompanied by NLRP3 activation, autophagy and increased lipid accumulation. However, supplementation with taurine (Tau) reduced MAFLD levels, possibly by reducing CTSB-dependent NLRP3 activation and pyroptosis [152]. In addition, lipid peroxidation acts as a bridge between autophagy and ferroptosis [153]. Park et al. found that: autophagy induces ferroptosis by degrading ferritin and inducing TFR1 expression; the ferroptosis inducers eastsin and RSL3 promote the assembly of autophagosomes and autophagy; and the inhibition of autophagy can induce intracellular iron consumption, reducing the occurrence of lipid peroxidation and ferroptosis [154]. However, Takamura et al. used autophagy-deficient mice to demonstrate that direct inhibition of autophagy leads to tumorigenesis [155]. Therefore, it may not be a useful therapeutic approach to directly inhibit autophagy. It may be better to indirectly regulate the related signaling cascade of upstream pathways, such as the AMPK/mTOR-related signaling pathway.

## Future directions: drugs targeting PCD to treat MAFLD

The pathogenesis of MAFLD is very complex, and thus far, there are no FDA-approved drugs on the market to treat this condition. Some fundamental molecular experiments have shown different degrees of therapeutic effects in MAFLD by targeting PCD. Further conversion into clinical applications is urgently needed.

The most widely used apoptosis-related pharmacological inhibitors, such as IDN-6556 and GS-9450, are being used in current clinical trials, and exciting results have been obtained (see Table 1 for details). Twenty-eight days of treatment with emricasan significantly reduced levels of ALT and CK-18, showing good safety and tolerance [156]. In addition, patients have been recruited for the phase IIb clinical trial of emricasan for MAFLD, with the primary outcome being improvement in fibrosis without MAFLD worsening (NCT02686762). The latest announcement said there was no statistically significant difference between all treatment groups and the placebo group (Table 2).

**Table 1:** The relationship between cell death and MAFLD

Name	Relationship with MAFLD
Apoptosis	Intestinal barrier dysfunction, oxidative stress, and ER stress lead to lipoapoptosis and activate the external death receptor pathway and internal mitochondrial pathway Glucose metabolism disorder activates the internal mitochondrial pathway and is regulated by several miRNAs
Necroptosis	Oxidative stress and intestinal barrier dysfunction trigger TNF-α-mediated necroptosis The key molecule RIPK1 is involved in the regulation of RIPK3 function and in the mutual transformation with apoptosis The interaction between RIPK3 and JNK is involved in disease progression, although the specific role is not clear Inhibition of MLKL improves insulin resistance, regulates fat metabolism, etc
Pyroptosis	Intestinal barrier dysfunction, ER stress, and oxidative stress all activate the assembly of NLRP3, and the secretion of inflammatory factors IL-1β and IL-18 leads to pyroptosis
Ferroptosis	Imbalance in the intracellular antioxidant system caused by excessive iron deposition and oxidative stress leads to disorders of the ferroptosis regulatory system and further affects lipid accumulation, inflammation, liver fibrosis, etc
Autophagy	Autophagy affects insulin resistance, fat metabolism, inflammation, liver fibrosis, etc. by regulating ER stress, oxidative stress, etc

**Table 2:** MAFLD-related programmed cell death markers and targets

Name	Contents	Refs.
Cytokeratin-18	It alone is not enough as a valuable biomarker, but it may have clinical significance when combined with other non-invasive methods of invasion, such as with serum adiponectin, serum resistin, uric acid (NCT01068444)	[18, 164, 165]
Fas/TNF-α	Inhibitor of death receptor-associated protein, including pentoxifylline, YLGA(Try-Leu-Gly-Ala) peptides	[35, 166]
Caspase enzymes	Inhibitor of apoptosis-related caspase enzymes, including VX-166, GS-9450 (NCT00740610), PF-03491390 (NCT02077374), and emricasan (NCT02686762, NCT02077374)	[34, 156, 157, 167]
R-3032	A ctsb inhibitor	[168]
Aramchol	Aramchol inhibits the liver enzyme stearoyl coenzyme A desaturase (SCD)	[25]
Selonsertib	ASK1 inhibitor	[169]
The farnesoid X-activated receptor (FXR)	Its agonist (NCT01265498) can improve serum transaminase levels in patients with MAFLD	[170]
Thioredoxin (TRX)	Oxidative stress can lead to a variety of PCD. TRX is induced by oxidative stress. Compared with healthy controls, the level of TRX rises significantly, but whether it can be used as a biomarker still needs further research	[171]
XIAP	XIAP antisense oligonucleotide (AEG35156) increases progression-free survival and overall survival	[172]
RIPK1/3	Although RIPK1/3 currently has inhibitors (GSKʹ840, GSKʹ843, GSKʹ872), according to different preclinical studies, it seems that directly targeting RIPK1/3 is not a better treatment for MAFLD, and further studies are needed	[173]
MLKL	Theoretically, the MLKL inhibitor (necrosulfonamide) may improve MAFLD, but evidence from well-designed clinical studies is still needed	[173]
NLRP3 inflammasome	Inhibiting the activation of the MAFLD inflammasome is an innovative treatment method, including an inhibitor of the NALP3 inflammasome (glyburide), a caspase 1 inhibitor (Pralnacasan), and an IL-1β antibody (canakinumab) or endogenous IL-1β inhibitor (anakinra)	[174]

Although the results of emricasan clinical trials for MAFLD are not ideal, it is undeniable that combined with the cascade reaction of caspase enzymes, the future research direction of direct pharmacological inhibitors targeting apoptosis may be more focused on inhibiting multiple targets or multiple PCD-related targets, due to the interaction between different PCD. A feasible strategy may be the selection of appropriate biomarkers, and then based on these, accurate and specific selection of drugs to achieve personalized treatment. Another effective method may be targeting pathways that regulate PCD, like AMPK/mTOR. In addition, the design of MAFLD clinical trial schemes, with unified diet and exercise management of subjects, accurate selection of clinical endpoints and so on, may improve the success rate of clinical trials.

In the MAFLD models induced by methionine–choline-deficient diets (MCDs) and high-fat diets (HFDs), apoptosis increased significantly, but VX-166 reduced caspase 3- and TUNEL-positive cells and decreased inflammation and other related indicators [157]. The caspase inhibitors IDN6556 and PF-03491390 reduce transaminase activity in patients with HCV [34, 158]. Pan-caspase inhibitors are currently available to study the clinical inhibition of apoptosis, whereas pharmacological targets for necroptosis are needed. Research shows considerable liver protection in MAFLD in the Ripk3^−/−^ genotype or with the use of dabrafenib, which inhibits RIPK3 kinase activity [159]. In addition, most pharmacological inhibitors that target pyroptosis concentrate on NLRP3, which reduces pyrolysis and the release of IL-1β and IL-18 and may have an improved effect on MAFLD. Inhibitory strategies for ferroptosis include directly inhibiting lipid peroxidation. Representative inhibitors of RTAs include α-tocopherol (α-TOH) [160], ferrostatin-1 (fer-1) [161] and liproxstain-1 (lip-1) [161]. The other method is to enhance Gpx4 activity by inhibiting ACSL4 and preventing activation of PUFAs for esterification to lysophospholipids by LPCAT3 [162] or by supplementing with nonoxidizing fatty acids, such as D-PUFA or MUFA [163].

It is worth noting that the role of autophagy in MAFLD seems to require further research. However, most current research drugs have both advantages and limitations for preclinical trial interventions and animal model studies of MAFLD. There are currently MAFLD models including dietary models, like HFD, MCD, choline-deficient l-amino acid-defined (CDAA); genetic models; and chemical models, like CCL4 and tetracycline. These can be easily analyzed to a certain extent and may partially reflect the disease characteristics and drug efficacy for MAFLD. However, due to the multiple complex pathological factors of MAFLD, these confounding factors are difficult to reproduce in animals. At the same time, there are some structural differences between animals and human beings and great differences in their MAFLD characteristics and drug response. Therefore, to assure the feasibility, safety and efficiency of therapy, the development of novel drugs for the treatment of MAFLD to target PCD should be mainly based on clinical experimental studies with better experimental designs as well as disease models for preclinical studies that can truly reflect the characteristics of human MAFLD.

## Conclusions

Over the past 10 years, our understanding of various types of programmed cell death has changed dramatically. Under normal physiological conditions, the various forms of programmed death play unique roles in maintaining the steady state of the normal body. However, when one or more of these processes is disrupted, disease may occur.

**Fig. 7:**
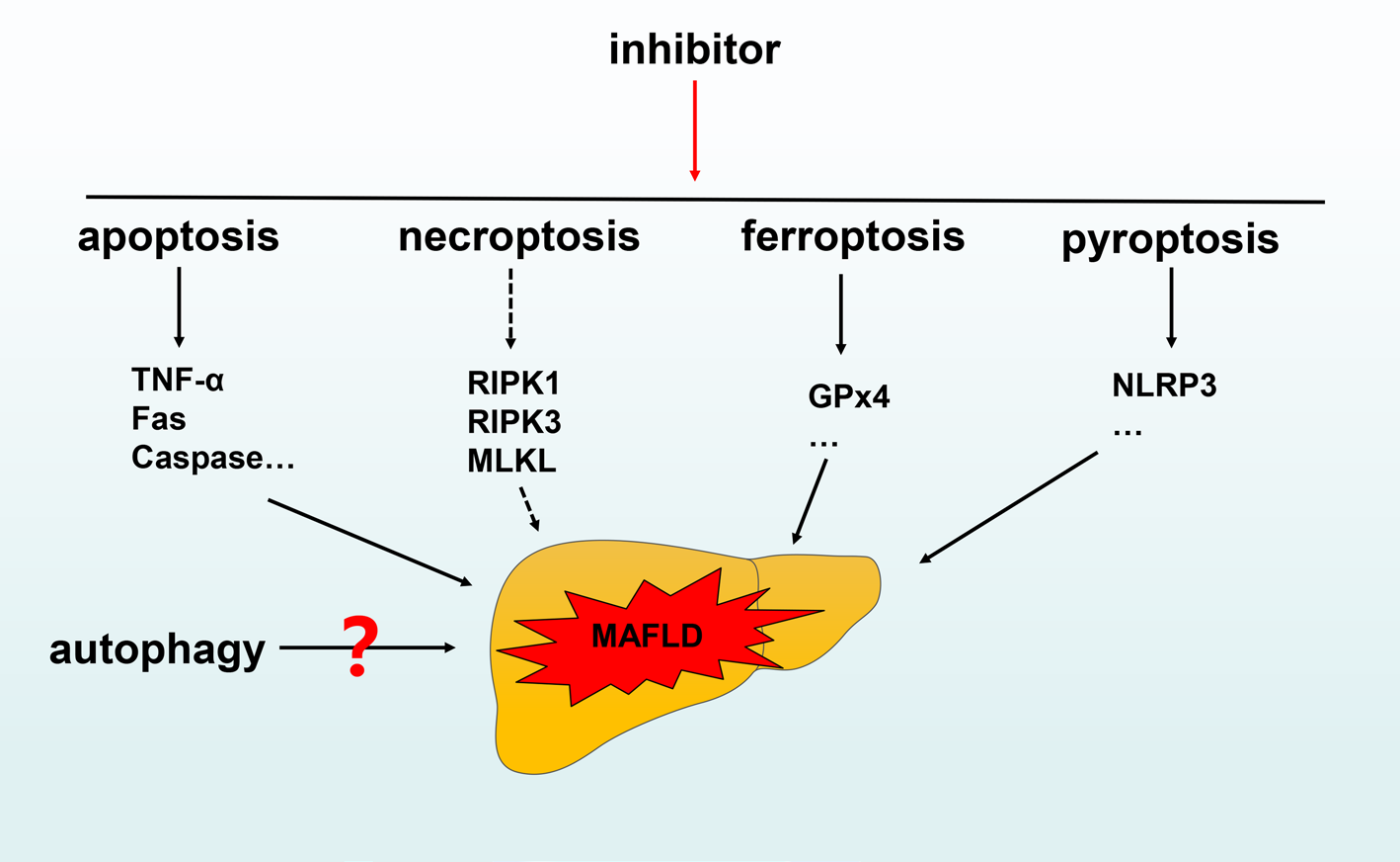
The relationship between programmed cell death and MAFLD. Inhibiting relevant targets in the apoptosis, ferroptosis and pyroptosis pathways may have therapeutic effects on MAFLD. In addition, there are various research results on targeting necroptosis and autophagy for treatment of MAFLD. Further research is needed

The pathogenesis of MAFLD is very complex. A variety of pathological factors, including oxidative stress and endoplasmic reticulum stress, activate various forms of programmed cell death and play key roles in this process. In other words, various pathogenic factors activate cell death programs. Therefore, the future direction of innovative treatment for MAFLD should include directly targeting activated cell death programs to improve disease, using compounds such as pan-caspase inhibitors (see Fig. 7 for details).

Here, we primarily reviewed and summarized the possible forms of programmed death in MAFLD, but there are still many issues that need to be addressed: for example, the specific interaction between the various forms of programmed cell death and their impact on disease. More importantly, to more accurately control the input and output of signals and accurately select one or more forms of death, we need a better understanding for targeting programmed death to treat MAFLD. This will provide a theoretical basis and guide future research.

## Data Availability

Not applicable.

## Abbreviations

NAFLD: Nonalcoholic fatty liver disease
MAFLD: Metabolic (dysfunction)-associated fatty liver disease
HCC: Hepatocellular carcinoma
CVD: Cardiovascular disease
RCD: Regulatory cell death
PCD: Programmed cell death
HCC: Hepatocellular carcinoma
MOMP: Mediates outer membrane permeabilization
CK-18: Cytokeratin-18
MCD: Methionine–choline-deficient diet
Grp78: Glucose-regulated protein-78
XBP-1: X-box binding protein-1
FGF21: Fibroblast growth factor 21
RYGB: Gastric bypass surgery
ERS: Endoplasmic reticulum stress
TNF: Tumor necrosis factor
TNFR: TNF receptor
BCL2: B cell lymphoma/leukemia-2
Bax: Accumulation of Bcl-2–associated X protein
Bak: BCL-2 homologous antagonist/killer
Bok: BCL-2 ovarian killer
Bad: BCL2-associated agonist of cell death
PUMA: BCL-2-binding component 3
BCL-XL: Also known as BCL2L1
BID: BH3-interacting domain death agonist
ROS: Reactive oxygen species
CHOP: ERS-related protein expression
Bim: (Also known as BCL2L11)
AAPC: Asiatic acid
JNK: C-Jun N-terminal kinase
SCD1: Stearoyl-CoA desaturase 1
LPS: Lipopolysaccharide
FFA: Free fatty acid
OSA: Obstructive sleep apnea
DPP4: Dipeptidyl peptidase 4
BP: Blueberries and probiotics
JAK1: Janus kinase 1
STAT3: Activators of transcription 3
CKIP-1: Casein kinase 2 interacting protein-1
IRS-1: Insulin receptor substrate-1
C3G: Cyanidin-3-O-β-glucoside
IHG: Intermittent hyperglycemia
MPT: Mitochondrial permeability transition
SIRT1: Sirtuin 1
UCDA: Ursodeoxycholic acid
CA: Creatine
PA: Palmitic acid
HFD: High-fat diet
DCA: Deoxycholic acid
PDCD4: Programmed cell death 4
RIPK1: RIP protein kinase family 1
RIPK3: RIP protein kinase family 3
MLKL: Pseudokinase mixed lineage kinase domain-like
CD95: CD95-type DR group
TRAIL: TNF-related apoptosis inducing ligand
FOXO1: Forkhead box protein O1
TLR4: Toll‐like receptor 4
TRIF: Toll IL-1 receptor (TIR) domain-containing adaptor-inducing interferon-β
ELA: Elafibranor
Nrf-2: Nuclear factor (erythroid-derived 2)-like 2
HO-1: Heme oxygenase 1
PIP3: Phosphatidylinositol (3,4,5)-trisphosphate
DNL: De novo lipogenesis
αGalCer: α-Galactosylceramide
PAMPs: Pathogen-associated molecular patterns
DAMPS: Damage-associated molecular patterns
NLRP3: NOD-LRR and pyrin domain-containing 3
ASC: Caspase-recruitment domain
IL-1β: Interleukin-1β
IL-18: Interleukin-18
IL-22: Interleukin-22
GSDMD: Cleaves gasdermin D
P2X7: Ligand-gated ion channel 7
UCP2: Uncoupling protein-2
GNP: Genipin
API: Apigenin
NLRP4: NOD-LRR and pyrin domain-containing 4 arsenic trioxide (AsO)
Tau: Taurine
AMPK: AMP-activated protein kinase
GPX4: Glutathione peroxidase 4
GSH: L-glutathione
L-glutathione: Phosphorylase kinase G2
PUFAs: Polyunsaturated fatty acids
SLC7A11: Solute carrier family 7 member 7
SLC3A2: Solute carrier family 3 member 2
FINs: Ferroptosis inducers
WA: Withaferin A
VDACs: Voltage-dependent anion channels
COA: Coenzyme A
IPP: Isopentenyl pyrophosphate
HSPB1: Heat shock factor protein 1 (HSF1)-heat shock protein b 1
Keap1: Kelch-like ECH-associated protein
AIFM2: Apoptosis-inducing factor mitochondrial
4-HNE: 4-Hydroxy-2,3-nonanal
MDA: Malondialdehyde
TRF1: Transferrin receptor 1
Nrf-2: Erythroid-derived 2)-like 2
DMT-1: Divalent metal transporter-1
ULK1: Unc-51-like kinase 1
ULK2: Unc-51-like kinase 2
mTORC1: Rapamycin complex 1
PE: Phosphatidylethanolamine
LC3: The microtubule-associated protein 1 light chain 3
Atg: Autophagy-related proteins
GNMT: Glycine N-methyltransferase
SAMe: S-adenosylmethionine
JAMD3: Jumonji-D3
SREBP-2: Sterol regulatory element-binding protein 2
PNPLA8: Phospholipase domain-containing enzyme 8
JZG: Jiang Zhi granule
TAK1: Transforming growth factor-β activated kinase-1
TRADD: TNFR1-associated death domain protein
TRAF2: TNF receptor-associated factor 2
TRAF5: TNF receptor-associated factor 5
cIAP1: E3 ubiquitin ligases cellular inhibitor of apoptosis 1
cIAP2: E3 ubiquitin ligases cellular inhibitor of apoptosis 2
LUBAC: Linear ubiquitin chain assembly complex
NEMO: NF-κB essential modulator
IKKβ: IκB kinase 2, also known as IKK2
IKKα: IκB kinase α
TAB2/3: TAK1 binding protein 2/3
HSC: Hepatic stellate cells
TGF-β: Transforming growth factor beta
UPR: Unfolded protein response
